# Interleukin 12 is associated with cerebral atrophy in childhood-onset systemic lupus erythematosus

**DOI:** 10.1186/1546-0096-12-S1-P334

**Published:** 2014-09-17

**Authors:** Mariana Postal, Aline Lapa, Karina Peliçari, Nailu Sinicato, Roberto Marini, Simone Appenzeller

**Affiliations:** 1 Medicine, State University Of Campinas, Campinas, Brazil

## Introduction

Cerebral atrophy has been described to occur in systemic lupus erythematosus (SLE) with variable frequency. Aging, corticosteroid use, inflammation and central nervous system (CNS) involvement may lead to cerebral atrophy. The higher sera levels of cytokines in childhood-onset SLE (cSLE) might induce cerebral alterations.

## Objectives

To determine the prevalence of cerebral atrophy in cSLE and to evaluate sera Th1 (IL-12, IFN-γ, TNF-α) and Th2 (IL-4, 5, 6 and 10) cytokines levels in cSLE. To elucidate the possible relationship between cerebral atrophy and Th1 and Th2 cytokine profile.

## Methods

We included consecutive cSLE followed at the pediatric rheumatology unit of the State University of Campinas were enrolled in this study. The control group was consisted by age and sex matched healthy individuals. A complete clinical, laboratory and neurological evaluation was performed in all subjects. Neurological manifestations were analyzed according to the ACR classification criteria. Mood and anxiety disorders were determined through Becks Depression and Becks Anxiety Inventory.SLE patients were further assessed for clinical and laboratory SLE manifestations, disease activity [SLE Disease Activity Index (SLEDAI)], damage [Systemic Lupus International Collaborating Clinics/American College of Rheumatology Damage Index (SDI)] and current drug exposures. Total dose of corticosteroids and other immunosuppressant medications used since the onset of disease were calculated by data obtained by careful review of the medical charts. MRI scans were performed in a 3T Phillips^®^scanner using a standardized protocol. Sagittal T1 weighted images were used for semiautomatic volumetric measurements. Volumes smaller 2 standard deviation from the means of controls were considered abnormal. Total dose of corticosteroids and other immunosuppressant medications used since the onset of disease were calculated by data obtained by careful review of the medical charts. Sera samples were obtained from all participants in the absence of infections. Th1 (IL-12, IFN-γ, TNF-α) and Th2 (IL-4, 5, 6 and 10) cytokines sera levels were measured by ELISA using commercial kits. Data were compared by non-parametric tests.

## Results

We included 76 cSLE patients (69 women; median age 16 years; range 9-30) and 66 (women 51; median age of 19 years; range 5-23) age and sex matched healthy controls. The median and range cerebral volume in patients with cSLE was 1067.9 (range 831.1-1449.2) cm^3^, compared with 1172.7 (range 941.9-1477.1) cm^3^ in healthy volunteers (p<0.001). Cerebral atrophy was identified in 18 (23.7%) cSLE patients and in 1 (1.5%) control (p<0.001). Significantly increased Th1 [IL-12 (p=0.016), IFN-γ (p<0.001), TNF-α (p<0.001)] and Th2 [IL-4 (p=0.046), 5 (p=0.013), 6 (p<0.001) and 10 (p=0.022)] levels were observed in SLE patients compared to controls. We observed an association between cerebral atrophy and IL-12 (p=0.034). We also observed an association between cerebral atrophy and aCL (p=0.023), cumulative corticosteroid dose/kg (p=0.024), dsDNA (p=0.024), anti-Sm antibodies (p=0.049) and depression (p=0.024).

## Conclusion

IL-12 was associated with cerebral atrophy in cSLE, suggesting immunological basis for global atrophy in SLE.

## Disclosure of interest

M Postal: None declared, A Lapa: None declared, K Peliçari: None declared, N Sinicato: None declared, R Marini: None declared, S Appenzeller Grant / Research Support from: FAPESP (2008/02917-0, 2011/03788-2, 2013/09480-5) CNPQ (300447/2009-4 and 471343/2011-0; 302205/2012-8; 473328/2013-5)

